# Analysis of related factors for RA flares after SARS-CoV-2 infection: a retrospective study from patient survey

**DOI:** 10.1038/s41598-024-52748-3

**Published:** 2024-02-20

**Authors:** Rong Li, Jun-Kang Zhao, Qian Li, Li Zhao, Ya-Zhen Su, Jun-yan Zhang, Li-Yun Zhang

**Affiliations:** 1 Third Hospital of Shanxi Medical University, Shanxi Bethune Hospital, Shanxi Academy of Medical Sciences, Tongji Shanxi Hospital, Taiyuan, 030032 China; 2 Department of Clinical Epidemiology and Evidence-Based Medicine, Shanxi Bethune Hospital, Shanxi Academy of Medical Sciences, Tongji Shanxi Hospital, Taiyuan, 030032 China

**Keywords:** Medical research, Rheumatology, Risk factors

## Abstract

SARS-CoV-2 and its variants are widely prevalent worldwide. With frequent secondary and breakthrough infections, immune dysfunction in RA patients, and long-term use of immune preparations, SARS-CoV-2 infection poses a significant challenge to patients and rheumatologists. Whether SARS-CoV-2 infection causes RA flares and what factors aggravate RA flares are poorly studied. A questionnaire survey was conducted on RA patients infected with SARS-CoV-2 after December 7, 2022, in China through a multicenter and inter-network platform regarding general personal condition, primary disease, comorbidity, SARS-CoV-2 vaccination, viral infection, and impact on the primary disease. A total of 306 RA patients were included in this study, and the patient data were analyzed, in which the general condition of RA patients, medication use before SARS-CoV-2 infection and post-infection typing and manifestations, and medication adjustment did not affect the Flare of RA patients after SARS-CoV-2 infection. The control of disease before SARS-CoV-2 infection (OR = 2.10), RA involving pulmonary lesions (OR = 2.28), and the recovery time of COVID-19 (OR = 2.50) were risk factors for RA flare. RA involving pulmonary lesions, control status of disease before infection, and recovery time of COVID-19 disease are risk factors for RA flare after SARS-CoV-2 infection.

## Significance & innovation section

RA patients have immune dysfunction, long-term use of immunosuppressive drugs, and low SARS-CoV-2 vaccination rate, which make them a vulnerable group during the COVID-19 pandemic.

Respiratory involvement, control status of disease before infection, and recovery time of COVID-19 disease are risk factors for RA flare after SARS-CoV-2 infection.

Controlling disease activity in RA patients, especially in the context of the SARS-CoV-2 pandemic, has positive implications.

## Background

Rheumatoid arthritis (RA) is an autoimmune disease characterized by chronic erosive arthritis involving multiple organs. The pathological basis is synovitis and pannus formation, which can lead to joint deformity and loss of function^1^. The disease course of RA is prolonged, multiple organs are involved, and patients are treated with corticosteroids, disease-modifying anti-rheumatic drugs, biological agents, and other immunosuppressive drugs for a long time, which impair immune function and make them more susceptible to viral infection^2,3^.

More than three years into the global pandemic, COVID-19 has posed a severe threat to human health and life. The frequent secondary infections and breakthrough infections in RA patients have become significant threats to global public health. A highly effective vaccine against SARS-CoV-2 offers hope that people can reduce their infection or ease the symptoms of COVID-19^5,6^. However, RA patients have the problems of poor vaccine response, delayed seroconversion, low conversion rate, and low effective inhibition effect^7,8^. In addition, the immunogenicity of the vaccine also has the possibility of causing the recurrence and aggravation of the original disease in RA patients. RA patients have not been included in the clinical development and phase III efficacy trials of some SARS-CoV-2 vaccines, and RA patients with active disease are not recommended to be vaccinated^9^.

RA patients are a vulnerable group during the COVID-19 pneumonia pandemic. Immunodeficiency and immunocompromised state caused by long-term use of corticosteroids or other immunosuppressive drugs are all high-risk factors for severe/critical COVID-19^10^. COVID-19 can lead to pneumonia, acute respiratory distress syndrome, renal failure, thrombotic complications, multi-organ failure, and death^11,12^. Multiple population-based or health system-based studies have shown that patients with RA are at increased risk of COVID-19 infection and are more likely to have adverse COVID-19 outcomes (a combined endpoint of hospitalization, mechanical support, or death)^13^. In addition, Rituximab is associated with an increased risk of COVID-19-related death and intensive care hospitalization or death. At the same time, whether glucocorticoids and Janus kinase (JAK) inhibitors can worsen the adverse outcomes of COVID-19 still needs to be further verified^14^.

With the emergence of Omicron variants with greater transmissibility, pathogenicity, and immune evasion, patients with RA face more significant crises and challenges. RA patients with immune dysfunction and long-term use of hormones and immunosuppressants may cause severe immune response and cytokine storm once infected with SARS-CoV-2^10,14^. In addition, the use of antiviral drugs may have a further adverse effect on the patient's disease status^15^. Although patients with rheumatoid arthritis (RA) are considered to be at high risk for COVID-19 and prone to adverse outcomes, there is no consistent consensus on the impact of COVID-19 infection on the disease and how to use hormonal and immunosuppressive therapeutics during infection. To clarify the above issues, this study designed a questionnaire for RA patients to collect data. Through multicenter and network platforms, a questionnaire survey was conducted on the general situation, clinical data, SARS-CoV-2 vaccination, and disease changes after infection of RA patients. To identify factors associated with Flare in RA patients infected with SARS-CoV-2. This research is the first study in China to investigate the changes in the real-world condition of RA patients infected with SARS-CoV-2, which is of great significance for guiding the clinical treatment of RA patients. In particular, managing RA patients in the context of COVID-19 provides a reasonable basis for clinicians and healthcare providers.

## Method

### Ethical approval

The study was approved by the Ethics Committee of Shanxi Bethune Hospital, and informed consent was obtained from each patient.

### Study design and participants

This study was a real-world survey in which information was collected from patients with RA first infected with SARS-CoV-2 in Shanxi Province and 27 other provinces in China between January and March 2023. The primary endpoint of this study was RA flare after infection with SARS-CoV-2. This study cites the consensus reached at OMERACT 10, where "RA flare" is defined as the worsening of signs and symptoms of sufficient intensity and duration to lead to a change in therapy. Meanwhile, the protocol also shows that Questionnaire survey is an effective method for assessing RA flares^16^. Patients with RA were recruited from rheumatology clinics and patient cohorts established by rheumatologists. All data collected were anonymized, original, valid, and assisted by rheumatologists. Patients who attended the outpatient department were provided with a paper-and-pencil questionnaire, which they completed under the guidance of rheumatologists, a process that typically took about 10 min. On the other hand, patients who took part in the online survey received a follow-up telephone consultation with rheumatologists, during which their condition was reassessed, and the provided information was confirmed. These measures ensure that the information we collect is true and reliable. The questionnaire involved 34 items, including demographic characteristics, clinical characteristics of RA, treatment, disease activity assessment, COVID-19 vaccination, COVID-19 infection, and treatment. For comprehensive details regarding the questionnaire, kindly refer to the appendix.

### Inclusion criteria

Patients were aged 18-80 years and diagnosed by rheumatologists according to the 2010 ACR/EULAR criteria.

Patients with the first infected by SARS-Cov-2

### Exclusion criteria

Questionaries with un-reasonable data

Questionaries with incomplete completion

**Figure 1:**
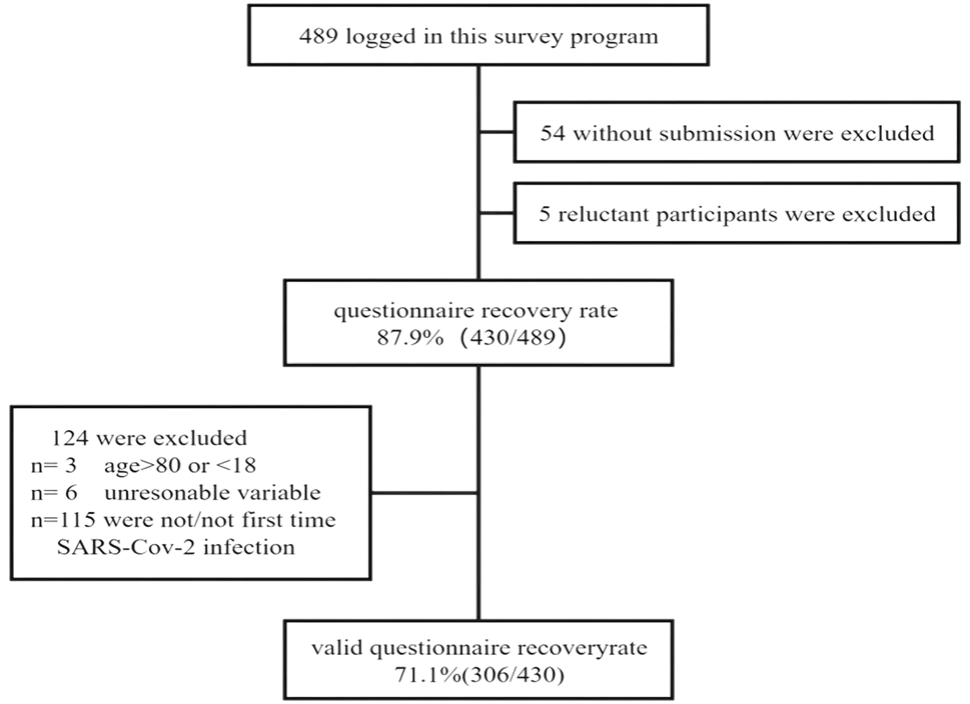
Process flowchart of questionnaire collection information.

The flow diagram is shown in flow Fig. 1.

### Outcomes

All outcomes and assignments were assessed by rheumatologists and reported by the patients. RA patients without apparent joint swelling and pain and typical inflammatory markers before COVID-19 infection were defined as the stable disease group, and the rest were the active disease group. According to the organ involvement of RA patients, they were divided into joint involvement, pulmonary lesions, and other system involvement. A total of 430 questionnaires were collected, of which 115 RA patients showed no signs of first SARS-CoV-2 infection, and 9 patients with abnormal data were excluded. This study included 306 patients with RA, including 242 outpatients and 64 online patients.

Statistical Analysis SPSS 22.0 software was used for statistical analysis. Continuous variables with normal distributions are presented as means and standard deviations. Continuous variables with abnormal distributions are continuous variables that were, respectively, presented as medians and interquartile ranges (IQRs). Baseline normal and abnormal quantitative data were analyzed using the student T-test and Wilcoxon rank-sum test. Categorical variables at baseline were analyzed using the chi-square test. Multivariate logistic regression analysis was performed for the outcomes analysis to identify the associations between dependent and independent variables. The results are expressed as adjusted odds ratios (ORs) with 95% confidence intervals (95% CIs). Significance was accepted as a two-sided test with an alpha level of 0.05.

### Ethical approval and consent to participate

This study was approved by the Ethics Review Committee of Shanxi Bethune Hospital and was conducted by relevant guidelines/regulations; Confirm that informed consent has been obtained from all participants and their legal guardians. And conducted research in compliance with the Declaration of Helsinki.

## Results

### Demographic and clinical data

Among the 306 patients, 262 were female, and 44 were male, with an average age of 51.44 ± 13.06 years. The disease duration ranged from 3 months to 32 years, averaging 115.67 ± 104.66 months. RA patients' affected organs and pre-infection medications (antiviral drugs, non-steroidal anti-inflammatory drugs, hormones, JAK inhibitors) were also described in accurate stratification (see Table 1 for details).

**Table 1:** Demographic characteristics, clinical characteristics and COVID-19 infection of patients with RA.

	Total (n = 306)	Inactive (n = 214)	Active (n = 92)
Sex (female), n (%)	262 (86)	183 (86)	79 (86)
Age (years)	51.44(13.06)	51.28(12.89)	51.82(13.52)
BMI (kg/m^2^)	23.18 (4.40)	22.99 (3.96)	23.61 (5.27)
Smoke, n (%)	20 (6.5)	11 (5.1)	9 (9.8)
Alcohol, n (%)	16 (5.2)	8 (3.7)	8 (8.7)
RA Course (months)	74.0(38.0,173.5)	70.5 (35.5,162.0)	100.0(38.3,229.3)
Comorbidities, n (%)	227 (74)	159 (74)	68 (74)
RA Organ involvement, n (%)			
* Only Arthrosis*	195 (64)	135 (63)	60 (65)
* Pulmonary system*	40 (13)	27 (12)	13 (14)
* Other systems*	68 (22)	47 (22)	21 (23)
Treatment of RA, n (%)			
* Glucocorticoid*	70 (23)	45 (21)	25 (27)
* Immunosuppressant*	247 (81)	175 (82)	72 (78)
* Biologics*	51 (17)	35 (16)	16 (17)
* JAK inhibitor*	49 (16)	34 (16)	15 (16)
COVID-19 vaccination, n (%)			
* No*	79 (26)	55 (26)	24 (26)
* Yes*	227 (74)	159(74)	68 (74)
COVID-19 Course(days)	12.24 (9.58)	11.62 (8.71)	13.77 (11.39)
COVID-19 Classified, n (%)			
* Asymptomatic*	7 (2.3)	6 (2.8)	1 (1.1)
* Mild*	269 (88)	187 (87)	82 (89)
* Moderate*	19 (6.2)	17 (7.9)	2 (2.2)
* Heavy and critical type*	11 (3.6)	4 (1.9)	7 (7.6)
Treatment of Covid-19, n (%)			
* Antiviral*	11 (3.6)	7 (3.3)	4 (4.3)
* NSAIDs*	179 (58)	130 (61)	49 (53)
* Oxygen therapy*	24 (7.8)	15 (7.0)	9 (9.8)
* Glucocorticoid*	22 (7.2)	12 (5.6)	10 (11)

Age, BMI, and COVID-19 Course are expressed using mean ± standard deviation. RA Course was expressed by median and quartile NSAIDs: Non-steroidal Anti-inflammatory drugs.

Comorbidities included hypertension, diabetes, coronary artery disease, respiratory disease. Immunosuppressive agents include: hydroxychloroquine, methotrexate, leflunomide, cyclophosphamide, cyclosporine, tacrolimus. Biological agents include: rituximab, TNF-ɑ inhibitors, IL-6 receptor antagonists, IL-17 inhibitors, belimumab, telitacicept.

The classification of the severity of COVID-19 is determined based on an evaluation of the patient's symptoms, clinical manifestations, and imaging of lung lesions, categorized as asymptomatic, mild, moderate, severe and critical. Due to the small number of moderate, severe, and critical RA patients included in this study, they were combined as moderate and severe. It is worth noting that nearly a quarter of RA patients are unvaccinated against SARS-CoV-2. Time to recovery from COVID-19 in RA patients (symptom resolution/negative nucleic acid test) and medication for COVID-19 treatment were also described (see Table 1 for details).

### Univariate analysis

Univariate analysis showed that the general conditions of RA patients (age, gender, BMI, smoking and drinking history, and whether they were complicated with other underlying diseases) and the clinical data of RA patients (course of disease, drug use) did not affect the flare after infection with SARS-CoV-2 (see Table 2 for details). The involvement of different organs in RA patients, especially the RA involving pulmonary lesions (OR = 2.26, 95% CI: 1.13–4.53), could aggravate the risk of Flare in RA patients infected with SARS-CoV-2.RA patients with poor disease control before SARS-CoV-2 infection (OR = 2.16, 95% CI: 1.30–3.61) and RA patients with prolonged recovery time from COVID-19 (OR = 2.35, 95% CI: 1.34–410) were more likely to have RA flare. Some RA patients were treated with oxygen therapy during hospitalization for COVID-19, and these patients generally had flare or aggravation (OR = 2.72, 95% CI: 1.13–6.67).

**Table 2:** Univariate and multivariate logistic analyses predicted changes in patients with RA.

	Univariable analysis		Multivariable analysis	
	OR (95% CI)	*P* value	OR (95% CI)	*P*-value
Sex (male)	1.03 (0.53–2.09)	0.935	1.26 (0.62 −2.68)	0.542
Age (above 45)	1.11 (0.66–1.90)	0.700	0.91 (0.52 -1.61)	0.748
BMI (above 24)	1.00 (0.60–1.65)	0.988		
Smoke	0.88 (0.35–2.41)	0.794		
Alcohol	2.14 (0.67–9.51)	0.242		
Comorbidities	0.97 (0.56—1.68)	0.902		
RA Course > 6 years	0.99 (0.61–1.60)	0.960	0.86 (0.51 -1.44)	0.566
RA Extra-articular involvement				
* Pulmonary system*	2.26 (1.13–4.53)	0.021	2.28 (1.09 -4.79)	0.028
* Other systems*	1.34 (0.73–2.43)	0.338	1.28 (0.67 -2.41)	0.447
Treatment of RA				
* Glucocorticoid*	1.55 (0.89–2.69)	0.121		
* Immunosuppressant*	0.92 (0.51–1.70)	0.778		
* Biologics*	0.76 (0.38—1.45)	0.413		
* JAK inhibitor*	1.13 (0.58—2.14)	0.702		
RA disease activity	2.16 (1.30–3.61)	0.003	2.10 (1.23 -3.58)	0.006
COVID-19 vaccination	0.77 (0.45–1.32)	0.337		
COVID-19 Course(days)	2.52 (1.39—4.57)	0.002	2.50 (1.33 -4.71)	0.004
COVID-19 Classified				
* Mild*	2.72 (0.46–51.88)	0.357		
* Moderate*	4.36 (0.58–91.10)	0.210		
* Heavy and critical*	7.20(0.83–161.88)	0.111		
Treatment of Covid-19				
* Antiviral*	1.78 (0.50–6.06)	0.350		
* NSAIDs*	1.29 (0.79 2.12)	0.311		
* Oxygen Therapy*	2.72 (1.13–6.67)	0.025	1.83 (0.70 -4.82)	0.213
* Glucocorticoid*	1.28 (0.52–2.99)	0.575		

### Multivariate logistic regression model

In the multivariate logistic regression model, RA involving pulmonary lesions (OR = 2.28, 95% CI: 1.09-4.79), disease control before infection (OR = 2.10, 95% CI: 1.23-3.58), and recovery time from COVID-19 (OR = 2.50, 95% CI: 1.09-4.79) were risk factors for flare in RA patients infected with SARS-CoV-2 (see Table 2 for details).

## Discussion

### Basic information on RA patients

In this Internet-based Questionnaire survey of RA patients, we found that the general conditions of RA patients, such as age, gender, alcohol/alcohol history, BMI, and medication status, were not associated with the disease changes of RA patients after SARS-CoV-2 infection. RA patients are more likely to have flares when their organs are involved, especially when RA involves pulmonary lesions. Similarly, among RA patients hospitalized for COVID-19, oxygen therapy was also a factor for flare in univariate analysis. The excessive activation of the immune system and the involvement of cytokines in destroying an alveolar structure are the key factors for severe lung disease in COVID-19 patients. Coronaviruses use membrane ACE2 receptors to enter human cells. At the same time, ACE2 is also highly expressed in lung cells of patients with RA-related interstitial lung disease (ILD), and patients with RA and ILD are more susceptible to high inflammation and acute lung injury than the general population^17,18^. In addition, RA-ILD and COVID-19-infected lung lesions have remarkable similarities in epidemiological, clinical, and immunological characteristics. This increases the difficulty in disease judgment, medication, and treatment of RA patients with lung involvement infected with SARS-CoV-2 and the risk of Flare in RA patients^19^. Some RA patients have multiple system involvement. Model 2 was established according to the system involvement, and RA patients were divided into joint involvement only, one organ involvement, and two or more organ involvement. The involvement of 2 or more organs was found to be a risk factor for RA flare, as detailed in the appendices.

### Drugs for the treatment of RA

The study found that the drugs used by RA patients before COVID-19 infection, such as hormones, DMARD, biological agents, and non-steroidal anti-inflammatory drugs, were not associated with flare. This is similar to the results of the current study. As a PRO derived from the real world, it verifies the safety of traditional synthetic DMARDs and biologic DMARDs in RA patients during ACOVID-19, which has important guiding significance for the clinical medication of RA patients^20^. Similar recommendations from the American College of Rheumatology and the European League Against Rheumatism in 2021 do not recommend abrupt discontinuation of corticosteroids, regardless of SARS-CoV-2 exposure, and patients should be maintained at a lower dose. For patients with asymptomatic or mild COVID-19 and relatively stable autoimmune disease, it is recommended to withhold immunosuppressive agents, biological agents (except tocilizumab), and JAK inhibitors (current evidence is controversial) until 7–14 days after COVID-19 symptom relief or 10–17 days from the date of nucleic acid positive^21,22^. For patients with severe or even life-threatening autoimmune diseases, hormones can be used based on weighing the pros and cons, and biological agents or JAK inhibitors can be initiated ^23^.

Baricitinib is an effective treatment for patients with COVID-19 pneumonia. By inhibiting the JAK1 and JAK2 pathways, it can block the immune cascade and reduce viral replication, which is beneficial for reducing respiratory failure and mechanical ventilation. It can also prevent the worsening of symptoms associated with COVID-19^24,25^. It has also been reported that using Baricitinib can increase the risk of critical illness of COVID-19 in RA patients. As a potent immunosuppressant, Baricitinib cannot distinguish between infected host cells and healthy cells and has an inhibitory effect on the immune response of patients during the treatment ^26^. In particular, RA patients have autoimmune dysfunction, and the use of JAK inhibitors (compared with TNF-α inhibitors) will increase the risk of infection, major adverse cardiovascular events (MACE), and thrombosis in RA patients^27^. Although JAK inhibitors have been widely used to treat COVID-19, the risk of preexisting Flare after SARS-CoV-2 infection in RA patients who use JAK inhibitors should also be vigilant. So. Clinicians should carefully consider the appropriate timing of JAK inhibitor administration in RA patients with COVID-19 pneumonia.

### Vaccination of patients with RA

Unvaccinated RA patients had a similar risk for RA flare after SARS-CoV-2 infection compared with patients with RA who were vaccinated against SARS-CoV-2. With existing studies showing that vaccination reduces symptoms of COVID-19, Kawano Y et al. reviewed outcomes in patients with Autoimmune rheumatic disease (ARD) who were infected with SARS-CoV-2 over the past years and found that the proportion of patients with ARD who had severe COVID-19 has decreased since the early days of the pandemic. However, unvaccinated ARD patients have more severe cases^28^. There is also evidence that the neutralizing titer of antibodies against the standard variant of S protein (D614G) in RA patients after vaccination is lower than that in the general population, and the effectiveness of vaccine prevention and the reduction of hospitalization is also lower^29–31^. Despite breakthrough infections in RA patients and the greater escape transmissibility of Micron and its variants, recent evidence suggests that immunity from vaccination and infection can reduce Omicron infectivity. Infectivity decreased markedly after breakthrough infection to 28% in vaccinated versus 36% in unvaccinated participants. The booster dose also reduces infectivity further, with an 11% reduction in the likelihood of transmission for each additional amount^32^.

Another concern, however, is that more than a quarter of RA patients have never been vaccinated because of disease activity or concerns about the safety of the SARS-CoV-2 vaccine, posing a potential risk for changes in patient condition. Existing studies have shown that vaccination of RA patients in remission or with low disease activity has no significant effect on disease activity or exacerbation. However, the COVID-19 vaccination rate in RA patients is still low^33–35^. The ACR has continuously issued updated guidelines for vaccinating RA patients, advocating that patients in stable condition receive the SARS-CoV-2 vaccine (optimal choice) and a booster dose. Vaccination is recommended for people at high risk as soon as possible, regardless of disease activity and severity (except in critically ill patients). For RA patients, the safety of inactivated vaccines (the safest), recombinant subunit vaccines, or mRNA vaccines is relatively high^36^. Therefore, clinicians should actively recommend SARS-CoV-2 vaccination in stable RA patients, and pre-exposure prophylaxis is an essential treatment for RA patients.

### Patients with RA and regular visits

RA patients have irregular return visits and self-withdrawal of drugs during COVID-19. The non-standard diagnosis and treatment methods aggravate the risk of Flare in RA patients to a certain extent. During the period of SARS-CoV-2 infection, one-third of RA patients reduced or discontinued immunosuppressive agents, and one-sixth of RA patients suspended or reduced the use of biological agents and small molecule targeted drugs. More than half of the patients did not have regular follow-up visits, and some patients adjusted their treatment drugs. Similarly, Adrian Ciurea et al. investigated the course management of RA patients in Switzerland during the COVID-19 pandemic. They found that the number of patient consultations decreased by 52%, and the proportion of drug non-adherence also increased by^37^. Regular medical visits and outpatient treatment (with antiviral drugs or monoclonal antibodies to treat SARS-CoV-2) can reduce the incidence of severe COVID-19 outcomes in ARD patients^38^. Clinicians should pay attention to the impact of COVID-19 on the medical treatment of RA patients, and more convenient medical methods such as online diagnosis and treatment and telephone follow-up can be carried out to ensure the medical needs of RA patients.

Medical insurance has a long-term significance for the disease control of RA patients. Zara Izadi et al. used the COVID-19 Global Alliance for Rheumatology (GRA) registry to extract data to observe global differences in COVID-19 pneumonia outcomes; It was found that social policies and resources (environmental pollution, national economy, government policies, health needs) had a large impact on COVID-19 outcomes of ARD patients globally^39^. Similarly, data from populations have found that the COVID-19 pandemic has disproportionately affected racial and ethnic minority groups, with higher mortality rates in African-American, Native American, and Latino communities^40^. Adequate medical insurance can reduce the risk of adverse outcomes in RA patients with COVID-19, and RA patients should actively seek medical care after being infected with SARS-CoV-2. Active management of SARS-CoV-2 exposure in RA patients should be based on known risk factors for poor prognoses, such as age > 65 years, comorbidities, and the degree of immunosuppression^41^.

### RA and COVID-19

Studies have found that RA patients with a long recovery time from COVID-19 are more likely to have RA disease exacerbation. The type and treatment of COVID-19 did not, which may also be related to the small number of critically ill cases of COVID-19 that were included in the study. COVID-19 and RA are highly similar in clinical manifestations (fever, myalgia, arthralgia, fatigue, etc.), pathogenesis, and treatment drugs. The complications, severity, and mortality of COVID-19 patients are caused by excessive activation of cytokines (IL-1, IL-6, TNF-a, CCL2, and CXCL10, etc.), namely cytokine storm, which is remarkably similar to the pathogenesis of immune disorders in RA patients^42,43^. In addition, cases of musculoskeletal manifestations due to COVID-19 immune disorders cannot be clearly distinguished from joint and/or muscle dysmenorrhea in RA patients.

RA disease and COVID-19 are mutual risk factors. SARS-CoV-2 induces a high inflammatory response of "cytokine storm" in RA patients through pleocytosis. Immune disorders and long-term use of hormones and immunosuppressants in RA patients are independent risk factors for SARS-CoV-2 infection and acute hospitalization of COVID-19^44,45^. In an earlier study from Wuhan, China, patients with ARD had an unusually high relative risk for SARS-CoV-2 infection compared with the general population (OR 10.90; 95% CI 5.43–21.89)^46^. Similarly, in the whole family, the infection rate was much higher in RA patients than in other family members (OR 2.68; 95% CI 1.14–6.27). ARD patients who also took hydroxychloroquine had a lower risk of COVID-19 than those who took other disease-modifying anti-rheumatic drugs (OR 01.0; 94% CI 0.044–19.1; *p* = 04.95)^47^. In addition to a higher risk of infection, RA patients have also been found to have a higher rate of hospitalization and risk of critical illness. National cohort studies in several countries have shown that ARD patients hospitalized within 30 days of COVID-19 diagnosis have a higher risk of ICU admission, acute renal failure requiring renal replacement therapy, and venous thromboembolism, as well as higher mortality compared with the general population^48,49^.

We also found that RA patients generally had poor sleep and psychological anxiety during COVID-19 pneumonia. Similarly, Dominic L. Sykes studied the lasting effects of COVID-19 on patients with ARD and found that long-term symptoms, including anxiety (*p* = 0.001), fatigue (*p* = 0.004), myalgia (*p* = 0.022), were common in patients with RA. In particular, women are more likely to have long-term residual symptoms^50^.

The symptoms of COVID-19 and RA are highly similar. SARS-CoV-2 infection with cytokine storm and immune system disorders increases RA patients' risk of disease activity. This study shows that involving pulmonary lesions, preinfectional disease control, and time to recovery from COVID-19 are risk factors for Flare in RA patients with SARS-CoV-2 infection. At present, hormones and anti-rheumatic drugs such as hydroxychloroquine, tocilizumab, and Baricitinib are used as the main treatment for COVID-19 infection. Clinicians should make the optimal choice by comprehensive evaluation when using drugs to treat COVID-19 and control RA disease activity.

However, there are still some limitations of our study, and recall bias is difficult to avoid by using the questionnaire to collect information. Due to the limitation of the people who filled in the questionnaires, the number of critical RA patients with COVID-19 was relatively small, and the death of RA patients due to COVID-19 was not included in this questionnaire. In the next step, we will use the national medical insurance system to collect data, taking the activity of the primary disease, organ involvement, and the use of hormones, immunosuppressants, and anticoagulants in RA patients in the year before and after December 2022 as the node (December 2022 in China can be regarded as not infected with SARS-CoV-2). Clinical and laboratory data were collected and analyzed. Various treatment methods' serological characteristics, efficacy, and safety were studied. The critical condition, mortality, and hospitalization expenses of RA patients were compared horizontally, and the short and long-term conditions of RA patients after COVID-19 infection were evaluated to provide more comprehensive guidance for the diagnosis and treatment of RA patients.

## Conclusion

Patients with RA are involved in lung lesions, disease control status before infection, and recovery time from COVID-19 disease, which are considered risk factors for RA outbreaks following SARS-CoV-2 infection.

## Consent for publication

The consent of the owner has been obtained and published.

### Supplementary Information

Supplementary Information.

## Data Availability

All raw data can be obtained by contacting the corresponding author.
