# Supplementary material for: Analysis of related factors for RA flares after SARS-CoV-2 infection: a retrospective study from patient survey
Source: Sci Rep. 2024 Feb 20;14:4243. doi: 10.1038/s41598-024-52748-3 (PMC10879520; doi:10.1038/s41598-024-52748-3)
Supplement: Supplementary file 1 — Supplementary Information. [file 41598_2024_52748_MOESM1_ESM.pdf]

yd 有道翻译 支持免费文档翻译240万字符/年 立即下载→ https://fanyi.youdao.com/download

Questionnaire on COVID-19 vaccination, infection and disease changes in patients with rheumatic immune diseases

Dear friends and family of patients with rheumatic diseases,

With the comprehensive liberalization of COVID-19 prevention and control policies, more and more patients have been infected with COVID-19, which has different degrees of impact on their original diseases. In order to provide a scientific basis for the diagnosis and treatment of rheumatic immune diseases, help people better relieve the pain and recover from healthas soon as possible, Shanxi Provincial Clinical Medical Research Center for Rheumatic Immune Diseases and Rheumatology Committee of Shanxi Medical Association jointly launched this questionnaire survey. This questionnaire is open to patients with rheumatic immune diseases all over the country. You are sincerely invited to participate in the questionnaire, which is expected to take 5 minutes. The questionnaire will be anonymous and your privacy will be strictly protected. If you agree to the survey, please choose to agree and fill out this questionnaire. Looking forward to your support, thank you!

Are you aware of the details of this survey and are you willing to participate in this survey? [Single choice] *

○ Yes, I understand and have volunteered for this investigation.

○ No (To the end of the questionnaire, the survey is over.)

Your contact number [Fill in the blank]

In order to follow up your condition changes and give reasonable diagnosis and treatment suggestions in time

____

Personal basic situation

1. Have you been diagnosed with rheumatic disease [single choice] *

Must be diagnosed by your doctor

○ Yes

○ None (Please skip to the end of the questionnaire and submit the answer)

1.1 Which of the following rheumatic diseases do you suffer from? [multiple choice] *

□ Rheumatoid Arthritis

□ Systemic lupus erythematosus

□ Sjogren's syndrome

□ Ankylosing spondylitis

□ Undifferentiated spondyloarthritis

□ Psoriasis and psoriatic arthritis

□ Systemic vasculitis

□ Behcet's disease

□ Scleroderma/systemic sclerosis

□ Hyperuricemia and gout

□ Adult onset Still's disease

□ Inflammatory myopathy (myositis/dermatomyositis)

□ Osteoarthritis

□ Antiphospholipid syndrome

□ Other ___________________

Depends on option 1 of question **3**

2. The city you currently live in: [Fill in the blanks] *

____

3. Your gender [single choice] *

○ Male

○ Women

4. Your date of birth: [Fill in the blank] *

____

5. Your height (CM) [fills up the topic] *

____

6. Your weight (kg)/fills up the topic

____

7. [single topic selection] * your credentials

○ Primary school and below

○ Middle school

○ High school

○ Junior college

○ Undergraduate degree

○ Graduate student or above

8. Your marital status [single choice] *

○ Married

○ Living together

○ Unmarried

○ Widowed

○ Divorced

9. Are you a current smoker [single choice] *

○ Yes

○ No

○ Quit smoking

10. Do you drink alcohol now [single choice] *

○ Yes

○ No

○ Quit drinking

Your rheumatic diseases before the COVID-19 infection

11. Date of your first diagnosis of rheumatic disease [Fill in the blank]

____

12. You have rheumatic disease control [single topic selection] *

○ Stable disease (no obvious symptoms of rheumatic diseases, no obvious abnormalities in laboratory tests such as erythrocyte sedimentation rate and C-reactive protein)

○ Active disease (obvious rheumatic symptoms, abnormal erythrocyte sedimentation rate, C-reactive protein, complement and other indicators)

13. Which organ systems are affected by your rheumatic disease? [Multiple choice] *

□ Joint

□ Lungs

□ Heart

□ Kidneys

□ Gastrointestinal tract

□ Nervous system

□ Blood

□ Skin

□ Other ________________

14. What medications for rheumatic diseases did you take before your COVID-19 infection? [Multiple choice] *

□ Glucocorticoids

□ Hydroxychloroquine

□ Methotrexate

□ Leflunomide

□ Cyclophosphamide

□ Mycophenolate mofetil

□ Cyclosporine

□ Tacrolimus

□ Rituximab (Rituximab, Hanlikang)

□TNF-α inhibitors (Humira, Handayuan, Cyprognide, Reike, Yisaipu, Enli, etc.)

□IL-6 Receptor antagonist (Amerol)

□IL-17 inhibitor (Kesanti)

□ Beliten, telitacept

□JAK inhibitors (tofacitinib, baritinib, upadacitinib)

□ Aspirin

□ Anticoagulants (low molecular weight heparin, nadroparin, warfarin, riarelto, etc.)

□ Ursodeoxycholic acid

□ Chinese herbs (Tripterygium wilfordii, total glucosides of paeony, etc.)

□ Other ___________________

15. In addition to rheumatic diseases, do you have any other chronic diseases? [Single choice] *

○ Yes

○ None

15.1 What other chronic diseases do you suffer from? [Multiple choice] *

□ Chronic heart disease, including congenital heart disease (excluding high blood pressure)

□ High blood pressure

□ Diabetes mellitus

□ High blood lipids

□ Cerebrovascular disease

□ Chronic lung disease (excluding asthma)

□ Asthma

□ Chronic kidney disease

□ Chronic liver disease

□ Chronic neurological diseases

□ Malignant tumor

□ Chronic blood diseases

□ AIDS /HIV infection

□ Other ________________

Depends on option 1 of question **18**

Your COVID-19 vaccination status and its impact on your rheumatic diseases

16. Number of COVID-19 vaccines (including inhaled vaccines) you have received [single choice] *

○0 (Please skip to question 25)

A. 1

A. 2

A. 3

A. 4

17. The type of COVID-19 vaccine you received is [multiple choice] *

□ Inactivated vaccine (BBIO, Wuhan Bio, Sinovac, etc.)

□ Adenovirus vector-based vaccine (Kangcino)

□ Recombinant subunit vaccine (Anhui Zifilong Kema)

□ Wantai Biological nasal spray vaccine

□ Fubitai mRNA COVID-19 Vaccine

□ Unclear

18. Date of your last COVID-19 vaccination [fill in the blank] *

____

19. After the new crown vaccine, you whether original rheumatoid disease recurrence or worse [single topic selection] *

It refers to the aggravation of existing symptoms, the appearance of new symptoms or abnormal laboratory indicators

○ No

○ Yes

19.1 Specific manifestations of recurrence or exacerbation [multiple choice questions] *

□ Worsening of preexisting symptoms

□ Laboratory tests are abnormal

□ The original drug was increased

□ Add new medications

□ Other ________________

Depends on choice 2 of question **23**

Your COVID-19 infection status and its impact on your rheumatic diseases

20. Have you been infected with SARS-CoV-2 since December 7, 2022? [Single choice] *

○ Yes (nucleic acid/antigen test positive)

○ Yes (no nucleic acid/antigen test but COVID-19 related symptoms)

○ Uncertain (no nucleic acid/antigen test and no COVID-19 related symptoms) (Please skip to the end of the questionnaire and submit your answer)

○ No (nucleic acid/antigen test negative) (Please skip to the end of the questionnaire and submit the answer sheet)

20.1 The date on which your nucleic acid/antigen test was positive or you developed symptoms related to COVID-19 [fill in the blank] *

____

Depends on the **25th** topic 1; 2 options

21. During your COVID-19 infection, which of the following symptoms have you had? [Multiple choice] *

□ No symptoms

□ Fever (maximum body temperature) ________________*

□ Muscle and joint pain

□ Chest tightness and shortness of breath

□ Heart palpitations

□ Fatigue

□ Headache

□ Sore throat

□ Stuffy, runny nose

□ Cough and sputum production

□ Changes in taste/smell

□ Diarrhea

□ Nausea and vomiting

□ Conjunctivitis

□ Other ________________

22. What measures have you taken to treat COVID-19 infection? [Multiple choice] *

□ Azvudine

□ Naimatvir tablets/Ritonavir tablets (Paxlovid)

□ Antipyretic drugs (ibuprofen, etc.)

□ Oxygen therapy

□ Hormones

□ Antibiotics

□ Baritinib

□IL-6 receptor antagonists (Amerol, etc.)

□ Traditional Chinese medicine (Lianhua Qingwen, etc.)

□ Other _________________

23. Your appetite during COVID-19 [single choice] *

○ Loss of appetite

○ Same as before infection

○ Increased appetite

24. Your sleep quality during COVID-19 [single choice] *

○ Very good

○ Better

○ Worse

○ Poor

25. Your average amount of sleep (hours) per night [fill in the blank] *

____

26. According to the following new champions league points type, which type you belong to? [Single choice] *

Mild: fever, cough, sore throat, headache, muscle pain, nausea, vomiting, diarrhea, loss of taste and smell, but no obvious shortness of breath, dyspnea or abnormal chest imaging

○ Mild type: obvious shortness of breath, dyspnea, and mild pulmonary lesions (inflammatory infiltration < 50%)

○ Severe: persistent worsening of clinical symptoms without relief, or severe lung lesions (inflammatory infiltration >50%) in imaging

○ Critical type: respiratory failure, septic shock, multiple organ dysfunction

○ Asymptomatic: nucleic acid or antigen positive but no symptoms of COVID-19

27. Have you been hospitalized for COVID-19? [Single choice] *

○ Yes

○ No

27.1 The number of days you were hospitalized [fill in the blank] *

____

Relies on the **33** topic the first option

28. Does COVID-19 aggravate symptoms of pre-existing rheumatic diseases? [Single choice] *

○ Yes

○ No (please skip to question 37)

28.1 What are the symptoms of aggravation [multiple choice] *

□ Chest tightness and shortness of breath

□ Heart palpitations

□ Joint swelling and pain

□ Skin and mucous membranes (rash, erythema, nodules)

□ Dry mouth and eyes

□ Oral/genital sores

□ Recurrent fever

□ Muscle aches

□ Muscle weakness

□ Headache and dizziness

□ Fatigue and weakness

□ Other ________________

29. Medical visits for pre-existing rheumatic diseases after COVID-19 infection [multiple choice] *

□ Hospital outpatient visits

□ Hospital admission

□ Telephone/Internet consultation

□ No consultation or medical treatment was given

29.1 The number of days you were hospitalized due to an exacerbation of your rheumatic illness [fill in the blank] *

____

The second option depends on the 37th

30. What medications for rheumatic diseases have you added or increased during your COVID-19 infection? [Multiple choice] *

□ None

□ Glucocorticoids

□ Hydroxychloroquine

□ Methotrexate

□ Leflunomide

□ Cyclophosphamide

□ Mycophenolate mofetil

□ Cyclosporine

□ Tacrolimus

□ Rituximab (Rituximab, Hanlikang)

□TNF-α inhibitors (Humira, Handayuan, Cyprognide, Reike, Yisaipu, Enli, etc.)

□IL-6 Receptor antagonist (Amerol)

□IL-17 inhibitor (Keshan ting)

□ Beliten, telitacept

□JAK inhibitors (tofacitinib, baritinib, upadacitinib)

□ Aspirin

□ Anticoagulants (low molecular weight heparin, nadroparin, warfarin, riarelto, etc.)

□ Ursodeoxycholic acid

□ Traditional Chinese medicine (Tripterygium wilfordii, total glucosides of paeony, etc.)

□ Other ________________

31. What medications for rheumatic diseases have you suspended or reduced during COVID-19? [Multiple choice] *

□ None

□ Glucocorticoids

□ Hydroxychloroquine

□ Methotrexate

□ Leflunomide

□ Cyclophosphamide

□ Mycophenolate mofetil

□ Cyclosporine

□ Tacrolimus

□ Rituximab (Rituximab, Hanlikang)

□TNF-α inhibitors (Humira, Handayuan, Cyprognide, Reike, Yisaipu, Enli, etc.)

□IL-6 Receptor antagonist (Amerol)

□IL-17 inhibitor (Kesanti)

□ Beliten, telitacept

□JAK inhibitors (tofacitinib, baritinib, upadacitinib)

□ Aspirin

□ Anticoagulants (low molecular weight heparin, nadroparin, warfarin, riarelto, etc.)

□ Ursodeoxycholic acid

□ Traditional Chinese medicine (Tripterygium wilfordii, total glucosides of paeony, etc.)

□ Other ________________

32. Your state of Mind during COVID-19 [single choice] *

○ The state of mind is relatively stable. If you are infected, you will be safe

There are some anxiety, panic, depression state, can be alleviated by self-regulation

○ Poor mental state, inability to self-regulate, even need professional intervention

33. Date of your recovery from COVID-19 [fill in the blank] *

If you meet one of these criteria, you are considered to be well: ①the patient's body temperature has returned to normal for more than 3 days; ②the symptoms basically disappeared or improved significantly; ③For patients with symptoms of pneumonia, the lesions of pneumonia were obviously absorbed on CT reexamination; ④Nucleic acid test negative for two consecutive days, or Ct value ≥35, or antigen negative for three times.

____

34. A message/fills up the topic

34. Do you have any difficulties or need any help with your rheumatic disease

____
